# The subtilisin-like protease SBT3 contributes to insect resistance in tomato

**DOI:** 10.1093/jxb/erw220

**Published:** 2016-06-03

**Authors:** Michael Meyer, Franziska Huttenlocher, Anna Cedzich, Susanne Procopio, Jasper Stroeder, Corinne Pau-Roblot, Michelle Lequart-Pillon, Jérôme Pelloux, Annick Stintzi, Andreas Schaller

**Affiliations:** ^1^Institute of Plant Physiology and Biotechnology, University of Hohenheim, 70593 Stuttgart, Germany; ^2^EA3900-BIOPI Biologie des Plantes et Innovation, Université de Picardie, 80039 Amiens, France

**Keywords:** *Manduca sexta*, pectin methylesterase, proteinase inhibitor, subtilase, systemin, wound signaling.

## Abstract

Subtilisin-like proteases (SBTs) constitute a large family of extracellular plant proteases, the function of which is still largely unknown. In tomato plants, the expression of *SBT3* was found to be induced in response to wounding and insect attack in injured leaves but not in healthy systemic tissues. The time course of *SBT3* induction resembled that of *proteinase inhibitor II* and other late wound response genes suggesting a role for SBT3 in herbivore defense. Consistent with such a role, larvae of the specialist herbivore *Manduca sexta* performed better on transgenic plants silenced for *SBT3* expression (*SBT3-SI*). Supporting a contribution of SBT3 to systemic wound signaling, systemic induction of late wound response genes was attenuated in *SBT3-SI* plants. The partial loss of insect resistance may thus be explained by a reduction in systemic defense gene expression. Alternatively, SBT3 may play a post-ingestive role in plant defense. Similar to other anti-nutritive proteins, SBT3 was found to be stable and active in the insect’s digestive system, where it may act on unidentified proteins of insect or plant origin. Finally, a reduction in the level of pectin methylesterification that was observed in transgenic plants with altered levels of SBT3 expression suggested an involvement of SBT3 in the regulation of pectin methylesterases (PMEs). While such a role has been described in other systems, PME activity and the degree of pectin methylesterification did not correlate with the level of insect resistance in *SBT3-SI* and *SBT3* overexpressing plants and are thus unrelated to the observed resistance phenotype.

## Highlight

The subtilisin-like protease SBT3 is induced as part of the wound response in tomato plants and contributes to defense against the specialist herbivore *Manduca sexta*.

## Introduction

Plant subtilases (SBTs) constitute a large family of mostly extracellular proteases of unknown function. Among SBTs are enzymes with relaxed substrate specificities that are thought to be responsible for non-selective protein turnover. Examples of these catabolic SBTs include cucumisin, an abundant protease in the juice of melon fruits (Kaneda and Tominaga, 1975; Yamagata *et al.*, 1994), and related enzymes from fruits and latices of many other plant species (Schaller *et al.*, 2012). SBTs also include proteases that are highly specific and are thus expected to contribute to the processing of selected target proteins by limited proteolysis at well-defined cleavage sites (Schaller *et al.*, 2012). The prime example for such a processing SBT is SBT6.1 from *Arabidopsis thaliana*. AtSBT6.1, the ortholog of mammalian site-1-protease (S1P), initiates the transduction of stress signals from the endoplasmic reticulum to the nucleus by specific cleavage and activation of bZIP transcription factors (Liu *et al.*, 2007a, b). Additional substrates of AtSBT6.1 include the precursor proteins of a peptide growth factor (Rapid Alkalinization Factor 23; Srivastava *et al.*, 2009) and pectin methylesterases (Wolf *et al.*, 2009). Processing by AtSBT6.1 occurs at canonical S1P cleavage sites, typically characterized by the amino acid motif RRXL or RXLX (with X representing any amino acid).

As another example of highly specific SBTs, phytaspases from tobacco and rice hydrolyse typical caspase substrates after the invariant aspartate residue, showing highest activity with the tetrapeptide VEID (Chichkova *et al.*, 2010). Like caspases in animal systems, phytaspases are involved in the regulation of programmed cell death, but their mode of action and physiological substrates are still unknown (Vartapetian *et al.*, 2011). This is true for the vast majority of plant subtilases including most of the 56 family members in Arabidopsis (Rautengarten *et al.*, 2005; Schaller *et al.*, 2012). Among the Arabidopsis subtilases, AtSBT1.1, AtSBT3.5 and AtSBT5.2 were implicated in the processing of phytosulfokines (Srivastava *et al.*, 2008), pectin methylesterase 17 (Sénéchal *et al.*, 2014a) and epidermal patterning factor 2 (Engineer *et al.*, 2014), but specific processing of any of these potential substrates by cognate subtilases remains to be shown in a physiological context.

As compared with the still rudimentary knowledge of their physiology, our understanding of structure and biochemistry of plant SBTs is quite advanced (Schaller *et al.*, 2012; Schaller, 2013). This is particularly true for cucumisin and tomato SBT3, which can be regarded as prototypical plant SBTs sharing the functional domains that are typically found in most of the family members (Ottmann *et al.*, 2009; Murayama *et al.*, 2012). In addition to the well-conserved subtilisin-like catalytic domain, they comprise a cleavable N-terminal signal peptide for targeting of the nascent polypeptide to the secretory pathway, a pro-domain acting as a potent inhibitor of its mature enzyme, the protease-associated (PA) domain typically found in plant SBTs as a large insertion between the His and Ser residues of the catalytic triad, and a C-terminal fibronectin III-like domain. Both enzymes undergo extensive post-translational modifications including glycosylation, disulfide bond formation, and proteolytic processing of the prodomain, which was shown to be an auto-catalytic process in SBT3 and a prerequisite for enzyme maturation and passage through the secretory pathway (Cedzich *et al.*, 2009; Ottmann *et al.*, 2009; Nakagawa *et al.*, 2010; Murayama *et al.*, 2012).

Mature SBT3 and cucumisin show similar substrate specificity, as both enzymes prefer basic peptide substrates with additional selectivity for the P1 and P2 residues (the two amino acids immediately upstream of the scissile bond; Schechter and Berger, 1967). However, the specific amino acid requirements in these positions differ for the two enzymes. SBT3 shows a strong preference for Gln at P1. While Gln is also accepted by cucumisin, Leu, Asn and particularly Met are also tolerated in this position. The P2 preference is Lys for SBT3 and Pro for cucumisin (Yonezawa *et al.*, 2000; Cedzich *et al.*, 2009).

PA domain function was revealed by crystal structure analysis and found to differ for the two enzymes. In cucumisin, the PA domain is located close to the active site channel and appears to contribute to substrate selectivity (Murayama *et al.*, 2012). In SBT3, on the other hand, the PA domain is required for dimerization and enzyme activity, as it keeps the active site channel open and accessible for potential substrate molecules (Ottmann *et al.*, 2009). However, structural modeling of representative Arabidopsis subtilases indicated that PA domain-mediated dimerization and enzyme activation are unlikely to be a general property of all plant SBTs, since residues that were found to be important for dimerization and an auto-inhibitory β-hairpin are only partially conserved in the Arabidopsis SBT family (Rose *et al.*, 2010). Consistent with this notion, the monomeric state was found to be the predominant form of cucumisin in solution (Murayama *et al.*, 2012).

The C-terminal fibronectin III-like domain is present in both enzymes and likely required to stabilize the catalytic domain (Schaller, 2013). Thermal stability is in fact remarkable for both enzymes, even more so for cucumisin, likely because of its more compact structure as compared with SBT3 (Ottmann *et al.*, 2009; Murayama *et al.*, 2012). Interestingly, both enzymes lack calcium, and the ability to maintain stability of the subtilisin fold in the absence of calcium appears to be a distinguishing feature of plant SBTs as compared with homologs from other organisms (Rose *et al.*, 2010).

In this study we investigate the physiological role of SBT3. Taking the leads from previous biochemical analyses, we addressed the hypothesis that SBT3 might be involved in plant defense against insect herbivores. Such a role had been suggested by the observations that (i) the wound signaling peptide systemin is cleaved by SBT3 (Cedzich *et al.*, 2009), (ii) the remarkable stability of plant SBTs that renders them insensitive to the adverse conditions within the insect’s digestive system (Chen *et al.*, 2005, 2007), and (iii) the ability of plant SBTs to modulate the activity of pectin methylesterases (PMEs; Wolf *et al.*, 2009; Sénéchal *et al.*, 2014a; Taurino *et al.*, 2014), which have been implicated in insect resistance (Körner *et al.*, 2009; Dixit *et al.*, 2013).

We report that the expression of SBT3 is induced by wounding and herbivory and that SBT3 expression levels correlate with resistance against tobacco hornworm larvae, a specialist herbivore on tomato. Addressing a potential involvement of SBTs in the control of PME activity and pectin methylesterification, we observed changes in cell wall composition in transgenic plants with altered SBT3 expression levels that, however, were not linked to the altered resistance phenotype. A potential role of SBT3 in systemin processing and wound signaling was supported by attenuated expression of systemic wound response genes, and may in part explain improved performance of insect larvae on plants silenced for *SBT3* expression. Alternatively, the defensive role of SBT3 may rely on its post-ingestive activity in the insect gut.

## Materials and methods

### Growth of tomato plants, wounding and insect bioassays

Tomato plants (cv. UC82B) were grown in the greenhouse at a 16-h photoperiod with supplemental light and a 26 °C/18 °C day/night temperature regime. Plants were fertilized at weekly intervals (GABI plus 12-8-11; N, P, K fertilizer at 2ml l^–1^). Experimental plants, as opposed to those that were grown for seed propagation, were excluded from phytosanitary procedures.

*Manduca sexta*, Johanson (Lepidoptera: Sphingidae) was cultivated as described (Bosch *et al.*, 2014b). Feeding assays on artificial diet (0.12g pellets of Gipsy Moth Wheat Germ Diet; MP Biomedicals; Eschwege, Germany) supplemented with test proteins (SBT3 or BSA, 100 µg g^–1^ fresh weight) were performed with freshly hatched *M. sexta* larvae. Food pellets were changed twice a day, and larval weight was determined on days 3, 6, 8, 9, and 10. Addressing herbivory-induced changes in gene expression, early fifth-instar larvae were allowed to feed on a single leaf of 2- to 3-week-old tomato plants for about 15min, until a leaf area of about 1.5cm^2^ had been consumed. Wounded and unwounded systemic leaves were harvested at the indicated time points and flash-frozen in liquid N_2_. The pooled leaf material of five plants was used for RNA extraction. For mechanical wounding, a hemostat was used to crush the terminal leaflet of a single leaf across the central vein. To analyse insect performance, 150 3-day-old *M. sexta* larvae were put on 6-week-old plants of each of the tomato genotypes. The plants were exchanged as needed when most of the leaf material had been consumed. The experiment was terminated when the first larvae reached the wandering stage.

### Transgenic tomato plants

For silencing of *SBT3* expression, a hairpin construct was generated (primer sequences are given in Supplementary methods at *JXB* online) comprising 219bp of the tomato (*Solanum lycopersicum*) *SBT3* cDNA (nucleotides 520–718; acc. AJ006376) in sense and antisense orientations in pHANNIBAL (Wesley *et al.*, 2001). The entire expression cassette comprising the cauliflower mosaic virus (CaMV) 35S promoter, the *SBT3*-hairpin and the OCS terminator was cut out with *Not*I and transferred into pART27 (Gleave, 1992). Transgenic tomato plants silenced for *SBT3* expression (*SBT3-SI* plants) were generated as described (Bosch *et al.*, 2014b). Stable integration of the transgene and independence of transformation events was confirmed by Southern blot analysis (see Supplementary Fig. S1B). Silencing of *SBT3* expression was confirmed at the transcript level by RT-PCR, and at the protein level by western blot analysis (Supplementary Fig. S2). Homozygous plants of the T3 generation were used in all experiments.

For *SBT3* overexpression, the open reading frame (ORF) was PCR-amplified from the *SBT3* cDNA and cloned into the *Sma*I and *Pst*I sites of pDH51 (Pietrzak *et al.*, 1986). The expression cassette comprising the CaMV 35S promoter, the SBT3 ORF and the 35S terminator was then moved from pDH51 into the *Eco*RI site of pRD400 (acc. U09365) and transformed into tomato plants as before (see Supplementary Fig. S1A). SBT3 expression levels were analysed by RT-PCR and western blot, and three independent *SBT3-OX* lines were chosen for further analysis (Supplementary Fig. S2). Homozygous plants from the T2 or T3 generation were used in all experiments.

The *SBT3* promoter:reporter gene construct was generated in pBI101 (acc. U12639) comprising a promoterless β-glucuronidase (GUS) cassette and the NOS-terminator in pBIN19. A 1956-bp PCR product amplified from the *SBT3* gene (Meichtry *et al.*, 1999; acc. AJ006380), upstream of and including the translational start codon was fused with the GUS ORF using the *Sal*I/*Bam*HI sites of pBI101. Tomato plants were transformed as before and four independent lines with single T-DNA insertions were confirmed by Southern blot analysis and used for further analysis (see Supplementary Fig. S3).

### RNA extraction and quantitative reverse transcription-PCR analysis

Samples of wounded and systemic tomato leaves were flash-frozen in liquid nitrogen and ground to a fine powder. Approximately 50mg of leaf material was extracted in 500 µl of peqGOLD Trifast (PEQLAB GmbH; Erlangen, Germany) according to the manufacturer’s instructions with an additional chloroform extraction step to increase RNA purity. RNA was quantified spectrophotometrically and only RNA with a 260/280nm ratio of 1.8 or higher was used for reverse transcription. RNA integrity was checked on 1% denaturing formaldehyde gels (for every fifth sample). Two micrograms of total RNA was used for first-strand cDNA synthesis with RevertAid reverse transcriptase (Thermo Scientific; Braunschweig, Germany) and random hexamer primers (Thermo Scientific). No RT controls (omitting the reverse transcription step) were performed on every seventh sample with reference gene primers to exclude genomic DNA contamination. cDNAs were diluted 1:10 in water and used for qPCR analysis. Tomato elongation factor 1α (*EF1α*, acc. no. X14449) and ubiquitin (*UBI3*; X58253) were used as internal reference genes and specific primer pairs were used to detect expression of *SBT*3 (AJ006376), *OPR3* (AJ278332), *LoxD* (U37840), *PI-II* (K03291), and *LapA* (U50151). Primer efficiencies and optimal primer concentration were determined experimentally. qPCR was performed with *Taq* DNA polymerase expressed in and purified from *E. coli* and SYBR-Green (Cambrex Bio Science Rockland Inc.; Rockland, ME, USA) in a Bio-Rad CFX Connect real-time PCR system (Bio-Rad; Munich, Germany) using 40 cycles of 95 °C for 30s, 62 °C for 30s, and 72 °C for 40s, followed by a melting curve protocol from 58 °C to 95 °C to confirm uniformity of PCR products. PCR reactions contained target gene primers at the indicated concentrations and 200 µM dNTPs in 3mM MgCl_2_, 20mM (NH_4_)_2_SO_4_, 0.016% Triton X-100, 2% DMSO, 50mM KCl, 10mM Tris/HCl pH 8.3, 0.08% Tween 20. For data analysis, the following equation (Pfaffl, 2001) was used to calculate the relative fold change in mRNA levels of target genes normalized against two reference genes:

$$\text{ratio}=\frac{E_{\text{target}}{}^{\Delta {\text{CP}}_{\text{target}}\left(\text{control}-\text{sample}\right)}}{E_{\text{ref}}{}^{\Delta {\text{CP}}_{\text{ref}}\left(\text{control}-\text{sample}\right)}}$$

The changes in mRNA expression are shown relative to the expression level in leaf material pooled from six wild-type plants prior to wounding.

### Northern and Southern blot analysis

For RNA gel blots, total RNA was extracted from tomato leaf samples (0.3g) using a phenol-based standard protocol. The RNA (4.5 µg) was separated on formaldehyde–agarose gels, and transferred to nitrocellulose membranes. For DNA gel blots, genomic DNA was isolated from tomato leaves using a standard cetyltrimethylammonium bromide (CTAB)-based extraction procedure. Ten micrograms of DNA were digested with the enzymes indicated in the respective figures, separated by agarose gel electrophoresis and transferred to nitrocellulose membranes. PCR-amplified fragments of the *SBT3* and *PI-II* cDNAs and the *nptII* (acc. number: YP_788126) gene were used as probes. RNA and DNA blots were hybridized with the radiolabelled probes and analysed on a phosphoimager as described (Schaller and Oecking, 1999).

### Alkalinization assay for systemin activity

The SBT3 overexpression construct was transformed into a *Solanum peruvianum* cell culture (kindly provided by Georg Felix and Thomas Boller) by particle bombardment as described previously (Cedzich *et al.*, 2009). Suspension cell cultures were established for selected cell lines and continuous measurements of extracellular pH were performed in 5ml of cultured cells 6–8 days after subculture (Schaller and Oecking, 1999). Synthetic systemin peptide (Pepmic; Suzhou, China) was added from a 1000-fold concentrated stock solution in water.

### Proteinase inhibitor assay

Four-week-old tomato plants were mechanically wounded with a hemostat across the midvein and a second time 1h later parallel to the midvein of the second and third primary leaflets. At each time point, the leaf material of five plants of each genotype (*SBT3-OX*, *SBT3-SI*, UC82B wild-type control) was harvested, weighed, frozen in liquid N_2_, and stored at –80 °C. The samples were ground in liquid N_2_ and total protein was extracted in 3ml extraction buffer (50mM Tris/HCl, pH 7.8; 7% (w/v) polyvinyl polypyrrolidone (PVPP); 1.67mM phenylthiourea; 0.3M KCl; 0.4mM ascorbic acid) per gram fresh weight. The extracts were cleared by centrifugation (16 000×*g*, 30min, 4 °C). Chymotrypsin (0.1mg ml^–1^ in 0.001M HCl; 100 µl) was added to 100 µl of the supernatant. After 10min at room temperature, residual chymotrypsin activity was analysed by addition of 1ml reaction buffer (66mM Tris/HCl, pH 7.8, 80mM CaCl_2_) and 300 µl *N*-benzoyl-L-tyrosine *p*-nitroanilide (1mg ml^–1^ in DMSO). The release of *p*-nitroaniline was monitored spectrophotometrically (Varian Cary 100 Bio; Agilent Technologies; Waldbronn, Germany) at 405nm. Plants were grown in fully randomized fashion and for each genotype and time point, three replicates (three groups of five pooled plants) were analysed.

### Stability of SBT3 in *M. sexta* frass

Frass (feces) was collected from fifth-instar *M. sexta* larvae raised on the three different tomato genotypes (*SBT3-SI*, *SBT3-OX*, UC82B). Samples (100mg) were ground in liquid N_2_ and extracted in 200 µl 50mM Tris/HCl pH 7.5, 100mM NaCl, 10mM β-mercaptoethanol, 0.5% (v/v) Triton X-100 and proteinase inhibitor mix (SERVA Electrophoresis GmbH, Heidelberg, Germany). Protein extracts were cleared by centrifugation (16 000×*g*, 10min, 4 °C) and analysed on western blots using an SBT3 antiserum as described (Cedzich *et al.* 2009), and by zymography as detailed below.

### Zymography

For extraction of midgut proteins, larvae were anesthetized with ethyl acetate, and a 1–2cm piece of the midgut was dissected. Protein samples were extracted as described above, but 1mM benzamidine, 0.01mM pepstatin A, 1mM EDTA and 0.1mM leupeptin were added instead of the commercial proteinase inhibitor mix. Samples were mixed with sample buffer (10% (v/v) glycerol, 50mM KOH/acetate pH 5.0, traces of methyl green) and separated by acidic native PAGE (http://wolfson.huji.ac.il/purification/Protocols/PAGE_Acidic.html, last accessed 30 May 2016). Gels co-polymerized with 0.5 % (w/v) gelatin were run for 10min prior to sample loading and then for 3h at 4 °C in 350mM β-alanine–140mM acetic acid at 80V with reversed polarity. Gels were washed in two changes of renaturation buffer (50mM Tris/HCl pH 7.5, 2.5% (v/v) Triton X-100 including proteinase inhibitors as above) and then incubated overnight with gentle agitation in the same buffer with 2% (v/v) Triton X-100. Proteinase activity was visualized by Coomassie Brilliant Blue R250 staining as cleared bands in a blue background.

### PME activity assay

PME activity was assayed as described (Klavons and Bennett, 1986) with minor modifications. Fifty milligrams of tomato leaf tissue ground in liquid N_2_ was incubated for 1h at 4 °C in extraction buffer (20mM Na_2_HPO_4_, 20mM citric acid, 1M NaCl, 0.1% (v/v) Tween 20, 0.2% (w/v) PVPP, adjusted to pH 7.0) under shaking. After centrifugation (16 000×*g*, 30min, 4 °C) the cleared supernatant was desalted by ultracentrifugation (10kDa MWCO, Vivaspin concentrators, Sartorius; Göttingen, Germany) using extraction buffer without PVPP and NaCl, and the protein concentration was determined. PME activity was assayed in a total volume of 300 µl reaction buffer containing 1 µg protein, 100 µg pectin from citrus fruit (≥85% esterified, Sigma-Aldrich; Taufkirchen, Germany), 0.025U alcohol oxidase (from *Pichia pastoris*, Sigma-Aldrich) in 50mM sodium phosphate buffer pH 7.5. After 30min at 28 °C, the reaction was stopped by the addition of the same volume of 2M ammonium acetate, 19.5mM acetylacetone and 49mM acetic acid and incubated at 68 °C for 15min. The absorbance was read at 420nm against a buffer-only blank to quantify PME activity as nmol methanol µg^−1^ protein min^−1^ using a reference curve of 0–175 nmol methanol.

### In-gel assay of PME activity

Cell wall-enriched protein extracts were prepared as described above, added to 2× loading buffer (40mM lysine, 40mM arginine, 30% (v/v) glycerol) and separated by isoelectric focusing on Ready Gel^®^ IEF Precast Gels (Bio-Rad) following the manufacturer’s recommendations. To visualize PME activity, the gel was washed for 30min in 25mM Tris/HCl pH 8.5, 5mM EDTA and then incubated for 30min in reaction buffer containing 20mM Tris/HCl pH 7.6, 5mM EDTA, 160mM NaCl and 1% (w/v) pectin from citrus fruit (>80% esterified). After two more washing steps in 20mM Tris/HCl pH 7.6, 5mM EDTA and 160mM NaCl, PME activity was detected by ruthenium red staining (0.01% (w/v); 15min) of de-methylesterified pectin.

### Analysis of cell wall composition

The degree of pectin methylesterification (DM) and cell wall sugar composition was analysed in 6-week-old *SBT3-OX*, *SBT3-SI* and wild-type (UC82B) plants. For each analysis, three leaflets from the second oldest fully developed leaf were pooled from six plants (7–10g fresh weight). Three biological replicates were analysed in duplicate for each of the three genotypes. The leaf material was ground in liquid N_2_ and lyophilized. For cell wall extraction (Carpita *et al.*, 2001), 100mg of lyophilized powder was heated twice to 70 °C in absolute ethanol for 15min and centrifuged. The pellet was solubilized in 1% (w/v) SDS in 50mM Tris/HCl pH 7.2 and heated to 70 °C for 30min. The cell walls were subsequently homogenized in phosphate buffer (100mM KH_2_PO_4_ pH 6.8). Aliquots were digested with amylase and lyophilized. Monomeric neutral and uronic sugars were analysed by high performance anion exchange chromatography (HPAEC). The cell wall digests were hydrolysed by 4M trifluoroacetic acid (100 °C, 4h), the trifluoroacetic acid was removed under nitrogen, and the digests were then diluted with ultrapure water to 1mg ml^–1^. Sugars were analysed on an ICS3000 system with pulsed amperometric detection (HPAEC-PAD) (Dionex, Thermo Fisher Scientific; Illkirch, France) equipped with a CarboPac PA-1 column (ID 4 mm×250mm) and guard column (ID 4 mm×50mm) run at 1ml min^–1^ and 30 °C column temperature. For neutral sugars the mobile phases were (A) H_2_O, (B) 160mM NaOH and (C) 200mM NaOH. Elution profiles were as follows: 0–25min 90% A and 10% B, 25–26min 0–100% C, 26–35min 100% C, 25–36min 100–0% C, 36–50min 90% A and 10% B. For uronic acids, the mobile phases were (A) 160mM NaOH and (B) 0.6M NaOAc in 160mM NaOH. Elution profiles were as follows: 0–5min 100% A, 5–35min 0–100% B, 35–40min 100% B, 40–42min 100–0% B and finally column re-equilibration in 100% A from 42 to 50min. The injection volume was 25 µl. The monosaccharides arabinose, fucose, galactose, glucose, rhamnose, xylose, galacturonic acid and glucuronic acid (Sigma-Aldrich) were used as standards.

DM was determined by quantification of methanol and acetate after saponification of pectin extracts. Pectins were dissolved in D_2_O (10mg ml^–1^), and a first ^1^H NMR experiment at 80 °C was performed on a Bruker Avance 300 spectrometer (Bruker BioSpin SA; Wissembourg, France) in order to check the absence of free methanol and acetate. Fifty-five microliters of NaOD (1M) in D_2_O was subsequently added into the NMR tube and a second ^1^H NMR spectrum was performed. DM values were calculated as described previously (Bédouet *et al.*, 2003) and expressed as a percentage per residue.

## Results and discussion

### Expression of SBT3 is induced by wounding and insect herbivory

**Fig. 1.**
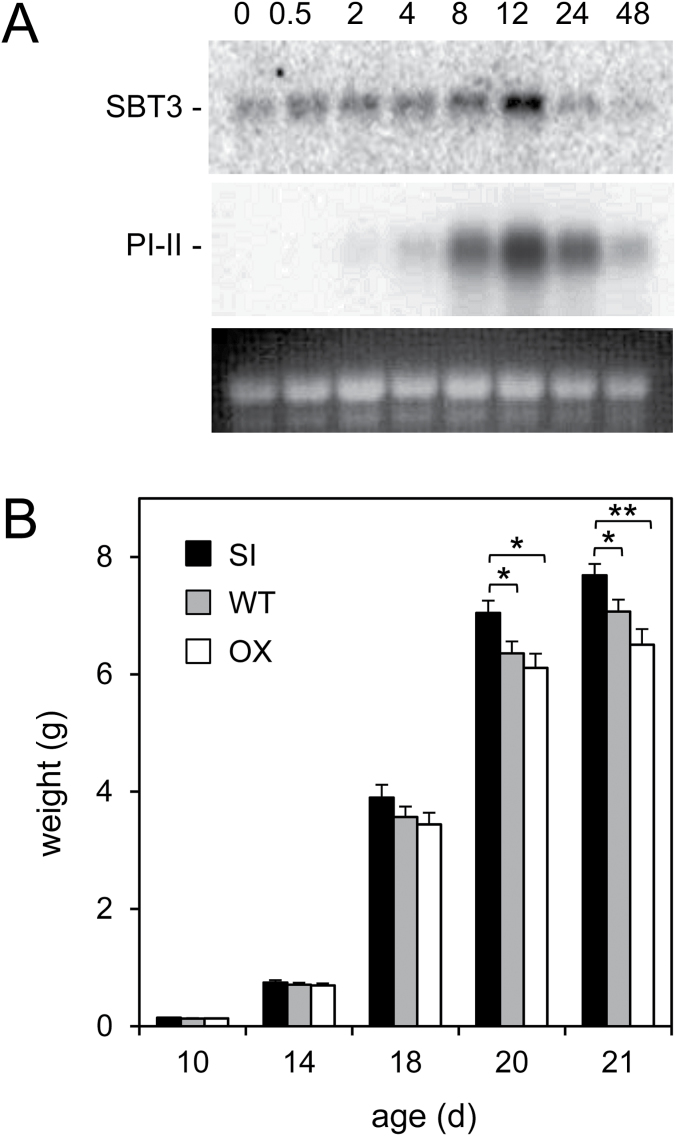
*SBT3* expression and its effect on insect performance. (A) RNA gel blot analysis of *SBT3* expression. RNA was isolated from leaves of tomato plants from 0 to 48h after the onset of *M. sexta* feeding, and 4.5 µg of total RNA were analysed on RNA gel blots using radio-labelled cDNA probes for *SBT3* (top) and *PI-II* (center). Blots were analysed on a phosphoimager (Typhoon Imager; GE Healthcare). A duplicated gel was stained with ethidium bromide as a control for RNA loading (bottom). (B) Effect of *SBT3* expression on the performance of *M. sexta* larvae. One hundred and fifty 3-day-old *M. sexta* larvae were placed on each of the three genotypes, *SBT3* over-expressors (OX, white bars), plants silenced for *SBT3* expression (SI, black bars) and wild-type controls (WT, gray bars). Larval weight is shown as the mean±standard error. Asterisks indicate statistically significant differences at *P*<0.05 (*) and *P*<0.01 (**); Mann–Whitney rank sum test.

**Fig. 2.**
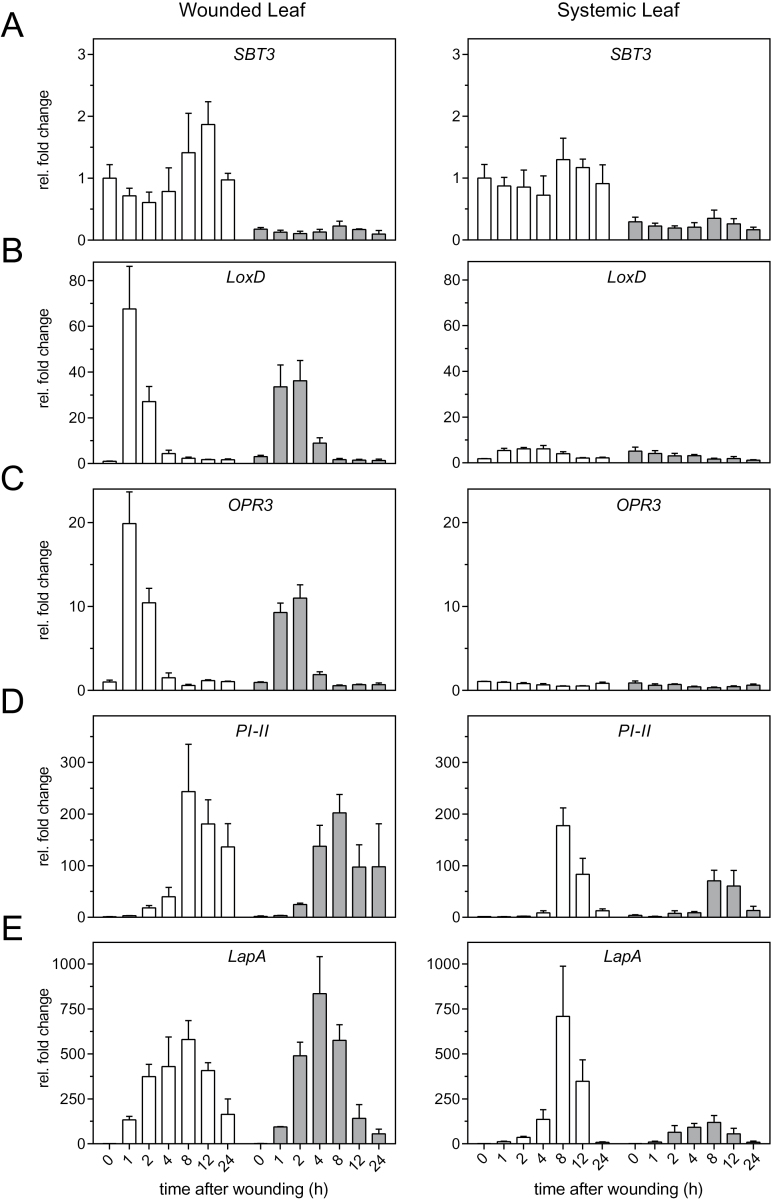
Local and systemic induction of *SBT3* as compared with early and late wound response genes. One leaf of 2-week-old wild-type (white bars) and *SBT3-SI* seedlings (grey bars) was wounded with a hemostat across the main vein of the terminal leaflet. At each time point after wounding, the damaged leaves (left) as well as the systemic unwounded leaves (right) of five plants were harvested and pooled for RNA extraction followed by qRT-PCR analysis. Transcript abundance of *SBT3* (A), two ‘early’ genes (B: *LoxD*, C: *OPR3*) and two ‘late’ genes (D: *PI-II*, E: *LapA*) was normalized to *UBI3* and *EF-1α* expression, and is given as fold change relative to healthy (0h) wild-type leaves. Data represent the mean±standard error of three biological replicates using three different *SBT3-SI* lines (SI lines 12, 14, and 21).

Addressing a potential function of SBT3 in herbivore defense, the expression of SBT3 and its response to the feeding of *M. sexta* caterpillars was analysed in leaves of tomato plants. On top of low-level constitutive expression, we observed a moderate but consistent increase in SBT3 mRNA abundance after insect feeding by northern blot analysis (Fig. 1A). Mechanical wounding resulted in a similar induction of the *SBT3* gene (Fig. 2A). The temporal pattern of *SBT3* induction was similar to that of *proteinase inhibitor II* (*PI-II*), a well-established marker for the ‘late’ wound response in tomato plants (Ryan, 2000). For the *PI-II* gene there was a strong increase after 8h and a maximum of expression 12h after the onset of insect feeding (Fig. 1A). Likewise after mechanical wounding, the kinetics of *SBT3* induction resembled those of *PI-II* and *Leucine Aminopeptidase A* (*LapA*), which was included as an additional late wound response marker (Fig. 2D, E). However, unlike *PI-II*, *LapA* and other systemic wound response genes that are induced in both the wounded and in distal unwounded leaves (Schaller *et al.*, 1995; Bergey *et al.*, 1996; Fowler *et al.*, 2009), systemic induction was not observed for *SBT3* (Fig. 2A).

As compared with *SBT3*, *PI-II* and *LapA*, much faster induction was observed for *lipoxygenase D* (*LoxD) and oxophytodienoate reductase 3* (*OPR3*), with highest transcript levels at 1 and 2h after wounding. These genes code for enzymes of the jasmonate (JA) biosynthetic pathway and were used here as markers for the early wound response (Ryan, 2000). Consistent with previous observations (Strassner *et al.*, 2002; Lee and Howe, 2003), there was very little, if any, induction of early genes in systemic leaves (Fig. 2B, C).

We conclude that the regulation of *SBT3* expression differs from previously described wound response genes. Slow induction in only the wounded tissue suggests that SBT3 activity may be required locally in late stages of induced herbivore defense in tomato.

### Insect resistance is reduced in plants silenced for SBT3 expression

In order to address a possible function in insect resistance, transgenic tomato plants were generated over-expressing *SBT3* under control of the CaMV 35S promoter (*SBT3-OX*), or silenced for *SBT3* expression by RNA interference (*SBT3-SI*). Over-expression and silencing of *SBT3* were confirmed at the transcript and protein levels in several independent transformants (see Supplementary Fig. S2), and three *SBT3-OX* (OX-2, -18, -19) and SI lines (SI-12, -14, -21) were chosen for further analysis. The transgenic plants had wild-type appearance and did not show any visible defects in growth or development.

One hundred and fifty first-instar *M. sexta* larvae were allowed to feed on each of the three genotypes, *SBT3-OX*, *SBT3-SI* and wild-type plants, and larval growth was followed for 21 days until they were ready to pupate and entered the wandering stage. Differences in weight gain were first noticed on day 10, and started to be statistically significant on day 20 (Fig. 1B). Larvae gained weight faster on *SBT3-SI* lines as compared with wild type, and more so when compared with *SBT3-OX* plants (Fig. 1B). Even though slower growth on *SBT3-OX* plants was observed repeatedly in several independent experiments, it was not statistically significant when compared with wild-type plants (Fig. 1B). However, the apparently reduced growth rate resulted in a longer time until pupation on *SBT3-OX* plants, with 11% entering wandering stage on day 21, as compared with 18 and 19% for those feeding on wild-type and *SBT3-SI* plants, respectively. A longer time until pupation increases the risk of predation in the field and is thus likely to affect the fitness of the herbivore (Feeny, 1976; Price *et al.*, 1980).

Enhanced performance of *M. sexta* larvae on plants silenced for SBT3 expression supports a role for SBT3 in plant defense. Addressing the specific function of SBT3 in insect resistance, three possible modes-of-action were investigated. (i) A role in defense signaling was suggested by the observation that SBT3 is able to cleave the wound signaling peptide systemin (Cedzich *et al.*, 2009). (ii) Pectin methylesterases (PMEs) have been implicated in insect resistance (Körner *et al.*, 2009; Dixit *et al.*, 2013). The recent finding of SBTs affecting PME activity (Wolf *et al.*, 2009; Sénéchal *et al.*, 2014a; Taurino *et al.*, 2014) thus suggested that changes in PME activity and pectin structure may be responsible for the insect resistance phenotype. Finally (iii), the remarkable stability of SBT3 and its high proteolytic activity at alkaline pH opened the possibility that the enzyme may exert its function only after ingestion, within the digestive system of the insect.

### Systemin processing

Wounding of tomato plants triggers the release of systemin, an 18-amino-acid signaling peptide, from its precursor protein prosystemin (Pearce *et al.*, 1991; McGurl *et al.*, 1992). Systemin is then perceived at the cell surface by a leucine-rich repeat receptor-like kinase and induces the expression of genes for jasmonate (JA) biosynthesis to amplify JA accumulation at the site of wounding as a prerequisite for systemic defense gene induction (Ryan, 2000; Wasternack *et al.*, 2006; Howe and Schaller, 2008). Systemin is a substrate of SBT3 *in vitro* and is cleaved specifically at Gln16, releasing the last two amino acids, Thr and Asp (Cedzich *et al.*, 2009). These two residues were shown to be important for receptor binding and activation (Meindl *et al.*, 1998; Scheer *et al.*, 2003), and C-terminally truncated systemin peptides are inactive with respect to the induction of PI-II accumulation (Pearce *et al.*, 1993). Cleavage by SBT3 therefore results in the inactivation of systemin suggesting a possible role for SBT3 as an attenuator of the wound response. Alternatively, if turnover of the peptide is required for continued signaling, cleavage by SBT3 might also augment the wound response.

**Fig. 3.**
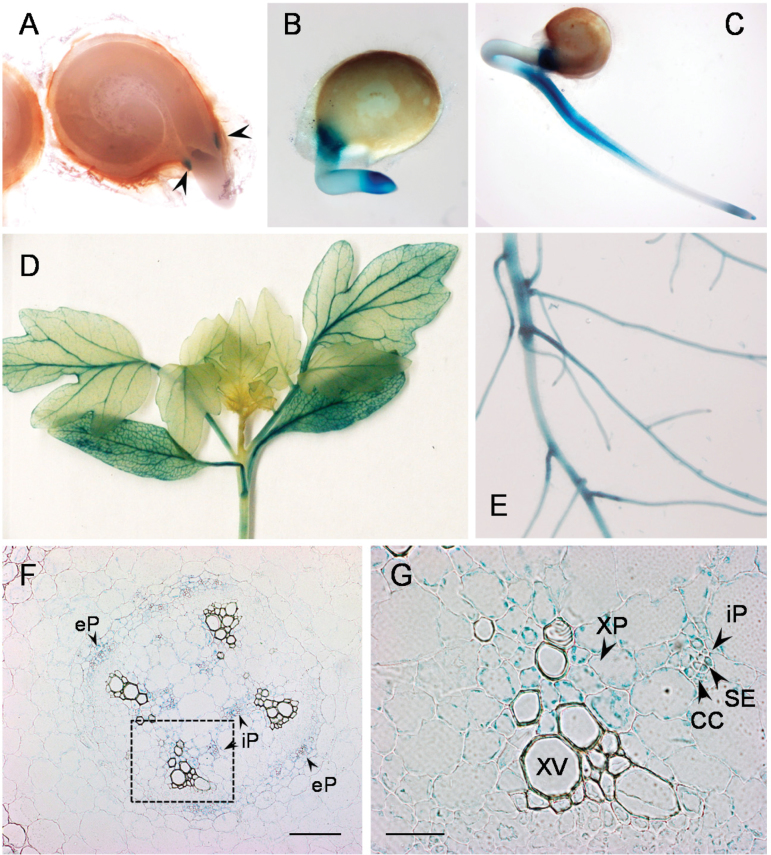
Promoter:reporter (GUS) analysis of SBT3 expression. A 2kb *SBT3* promoter fragment was used to drive the expression of the β-glucuronidase (GUS) ORF in transgenic tomato plants. GUS activity was analysed histochemically in seeds at 24h (A), 48h (B), and 72h (C) after imbibition, in shoots (D) and roots (E) of 3-week-old plants and in cross sections of the stem (F, light microscopy, with inset enlarged in G). Arrow heads in A: micropylar endosperm. Scale bars: 200 µm in F, 30 µm in G. CC, companion cell; eP, external phloem; iP, internal phloem; SE, sieve element; XP, xylem parenchyma; XV, xylem vessel. Equivalent expression patterns were observed in four independent transgenic lines.

Any function related to systemin processing would require co-localization of SBT3 and its putative substrate *in vivo*. Promoter:reporter (GUS) analysis was thus performed in transgenic tomato plants to assess the tissue-specific expression of *SBT3*. *SBT3* promoter activity was first detected during early seedling development in the micropylar endosperm as well as in the developing root (Fig. 3A–C). GUS expression was also detected in the mature root system, particularly at the junction between primary and lateral roots (Fig. 3E). Potentially relevant with respect to systemin signaling is the expression in the shoot vasculature (Fig. 3D). GUS staining was observed in both external and internal phloem including sieve elements and companion cells, and also in the vascular (xylem and phloem) parenchyma (Fig. 3F, G). Interestingly, this is where the early wound response pathway appears to be located. The prosystemin gene is expressed in vascular bundles (Jacinto *et al.*, 1997) and the protein was localized in parenchymatic cells of the phloem (Narváez-Vásquez and Ryan, 2004). Also present in the vasculature are the enzymes contributing to JA biosynthesis. Allene oxide synthase and allene oxide cyclase were located in the vascular parenchyma, companion cells and sieve elements of tomato stems, petioles and flower stalks (Hause *et al.*, 2000, 2003). The apparent co-localization of SBT3 with the early wound response pathway in the tomato vasculature would be consistent with SBT3 being involved in systemin processing and wound signaling.

**Fig. 4.**
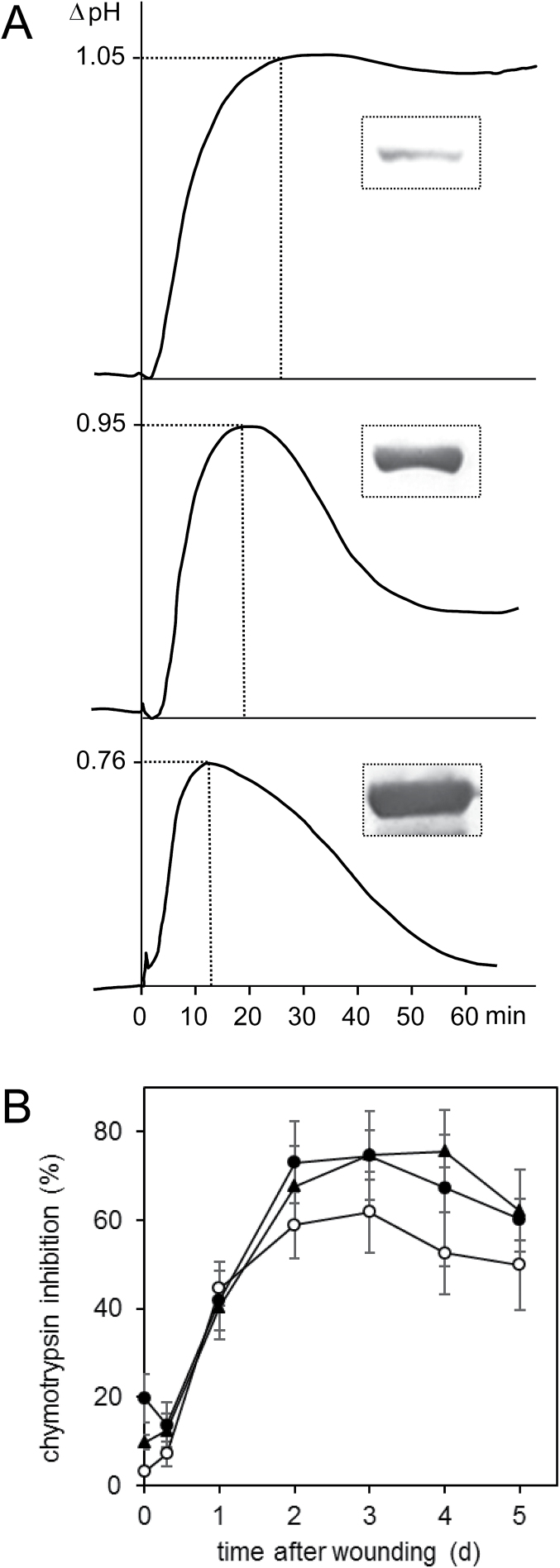
Effect of SBT3 expression on early and late systemin responses. (A) Systemin-triggered alkalinization response. Medium pH was recorded in tomato (*S. peruvianum*) cell cultures after addition of 1nM systemin at *t*=0min. The alkalinization response was compared in three independent transgenic cell lines showing different levels of SBT3 expression as indicated by the western blot signal. (B) Induction of proteinase inhibitor activity in response to wounding. The inhibition of chymotrypsin activity after addition of plant extracts obtained from *SBT3-OX* (line G2, triangle), *SBT3-SI* (line 21, filled circles) and wild-type plants (open circles) at the indicated time points after wounding was analysed in triplicate (three biological replicates, each including the pooled leaf material of five plants) and is shown as the mean±standard deviation.

To address a potential role of SBT3 in the regulation of systemin activity we looked at early and late systemin responses in transgenic plants and cell cultures exhibiting different levels of SBT3 expression. Among the earliest cellular responses to systemin is the depolarization of the plasma membrane, which was found to be necessary and sufficient for the activation of downstream defense gene expression (Schaller and Oecking, 1999; Schaller and Frasson, 2001; Maffei *et al.*, 2004; Mousavi *et al.*, 2013). Concomitant ion movements include the influx of H^+^ and Ca^2+^ and the efflux of K^+^ and Cl^–^, resulting in the alkalinization of the extracellular space that can be measured conveniently in cell suspension cultures by continuous recordings of medium pH (Felix and Boller, 1995; Schaller, 1998). In tomato (*S. peruvianum*) cell cultures, systemin-triggered medium alkalinization is dose-dependent and saturated at concentrations above 1nM (Vetsch *et al.*, 2000). The response reaches its maximum within 15min after addition of the peptide resulting in a pH increase of 0.8–1 (Vetsch *et al.*, 2000). Systemin-triggered medium alkalinization was reduced in both amplitude and duration in transgenic cell cultures expressing increasing levels of SBT3 (Fig. 4A). The duration of the alkalinization response was previously shown to depend on the metabolic stability of the peptide inducer (Schaller, 1998). The correlation that was observed between SBT3 expression levels and the attenuation of the alkalinization response (Fig. 4A) is thus consistent with systemin being cleaved by SBT3 in the cell culture system.

In order to address a potential effect of the SBT3 expression level on the output of the systemin signaling pathway, the expression of early and late wound response genes was compared in leaves of S*BT3-SI* and wild-type tomato plants in a time series after wounding (Fig. 2). The transcripts of early (*LoxD*, *OPR3*) and late (*PI-II*, *LapA*) wound response genes showed similar induction kinetics and levels in the leaves of wounded *SBT-SI* and wild-type plants (Fig. 2, left panels). We also analysed the induction of proteinase inhibitor activity over an extended time period, which reached its maximum after 2 days in wounded leaves with no significant difference between *SBT3-OX* and *SBT3-SI* transgenics as compared with wild-type tomato plants (Fig. 4B). Therefore, despite the apparent co-localization of SBT3 and prosystemin in the tomato vasculature (Fig. 3), and the ability of SBT3 to attenuate early responses to systemin treatment in the cell culture system (Fig. 4A), the local wound response was not affected by SBT3.

In contrast to the intact local response, systemic induction of late genes was clearly impaired in *SBT3-SI* plants (Fig. 2, right panels). Eight hours after wounding *PI-II* transcripts were induced 70-fold in systemic *SBT3-SI* leaves, as compared with 180-fold induction in corresponding wild-type leaves. Similarly, the systemic induction of *LapA* transcripts at 8h after wounding was much lower in *SBT3-SI* (120-fold) than in wild-type plants (710-fold; Fig. 2D, E). The data indicate that SBT3 function is dispensable for the local response, while it is needed to achieve full induction of defense genes in systemic leaves.

Whether or not the apparent role of SBT3 in the systemic wound response depends on its ability to cleave systemin remains to be shown. Considering the late induction of *SBT3* expression as compared with genes for JA biosynthesis (*LoxD*, *OPR3*), alternative functions downstream or independent of JA production seem more likely, e.g. for the exit of the systemic signal from the wound site, or as a suppressor of a negative regulator in systemic leaves. A JA-independent function in wound signaling has previously been described for LapA, for which the mechanism of action is also still unknown (Pautot *et al.*, 1993; Fowler *et al.*, 2009). Similar to what we observed for *SBT3-SI* plants, wound-induction of late genes is reduced in LapA-deficient plants, while the expression of early wound-response genes is unaffected. However, in contrast to SBT3, LapA affects both the local and the systemic induction of *PI-II* and other late genes (Fowler *et al.*, 2009). Consequently, the impact on larval development is much stronger in *LapA-SI* (Fowler *et al.*, 2009) as compared with what we observe in *SBT3-SI* plants (Fig. 1B). The stronger resistance phenotype in *LapA-SI* as compared with *SBT3-SI* plants is also consistent with recent finding showing that the local wound response is sufficient for defense against *M. sexta*. Comparing larval growth and development on wild-type tomato and transgenic plants impaired only in systemic signaling, it was found that defense gene induction in systemic tissues is not required to maintain wild-type levels of resistance against *M. sexta* (Bosch *et al.*, 2014*b*).

We conclude that SBT3 affects the systemic wound response, which is not necessarily related to its ability to cleave the wound signal systemin. The reduced expression of defense genes in systemic tissues of *SBT3-SI* plants may explain the reduced resistance phenotype observed in these plants. However, because of the limited relevance of systemic defense responses for defense against *M. sexta* (Bosch *et al.*, 2014*b*), alternative possibilities should be considered.

### Pectin methylesterase activity and cell wall composition

Pectin methylesterases catalyse the de-methylesterification of homogalacturonan (HG), the major pectin constituent of the primary cell wall (Sénéchal *et al.*, 2014*b*). Partially de-methylesterified (demethylated) HG may bind Ca^2+^ resulting in the formation of pectin gels increasing the rigidity of the cell wall. Alternatively, the cell wall may be weakened when partially demethylated HG is degraded by polygalacturonases or pectate lyases (Pelloux *et al.*, 2007). In either case, the demethylation of HG by PMEs has dramatic consequences on the mechanical properties and digestibility of the cell wall (Peaucelle *et al.*, 2011) and is thus likely to affect the performance of herbivorous insects (Körner *et al.*, 2009; Calderón-Cortés *et al.*, 2012).

In addition to changing cell wall mechanics, the demethylation of HG by PMEs releases substantial amounts of methanol, which may affect plant–insect interactions in multiple ways as it is directly toxic to larvae and may serve as a signal for direct and indirect plant defense responses (Körner *et al.*, 2009; Dixit *et al.*, 2013; Hann *et al.*, 2014; Komarova *et al.*, 2014; Sénéchal *et al.*, 2014*b*). Indeed, insect feeding was reported to induce PME activity and methanol emission (Penuelas *et al.*, 2005; von Dahl *et al.*, 2006), and *M. sexta* larvae show a small but consistent increase in performance on transgenic plants silenced for PME expression (Körner *et al.*, 2009), whereas on PME-overexpressing tobacco, the development of polyphagous insects is severely impaired (Dixit *et al.*, 2013). PME activity thus appears to be positively correlated with insect resistance in tobacco. Interestingly, PME activity is controlled in part by SBTs and, therefore, SBTs may be expected to exert an indirect effect on insect resistance by modulating PME-mediated methanol emissions and cell wall composition.

Both positive and negative effects of SBTs on PME activity have been described. Type-I/group 2 PMEs are synthesized as inactive pre-pro-enzymes and SBTs (Arabidopsis SBTs 3.5 and 6.1) were found to be required for the processing of the inhibitory prodomain and secretion of mature PMEs into the cell wall (Pelloux *et al.*, 2007; Wolf *et al.*, 2009; Sénéchal *et al.*, 2014*a*). Arabidopsis SBT1.7, on the other hand, appears to be involved in PME inactivation or degradation. In seeds of the SBT1.7 loss-of-function mutant, PME activity is increased with a concomitant reduction in HG methylation of seed mucilage, resulting in a failure to release mucilage upon hydration and a germination defect under low-water conditions (Rautengarten *et al.*, 2008). A similar phenotype was reported for a PME inhibitor (PMEI6) mutant, and the importance of AtSBT1.7 and the inhibitor for down-regulation of PME activity and mucilage release was confirmed by the additive phenotype of the double mutant (Saez-Aguayo *et al.*, 2013).

**Fig. 5.**
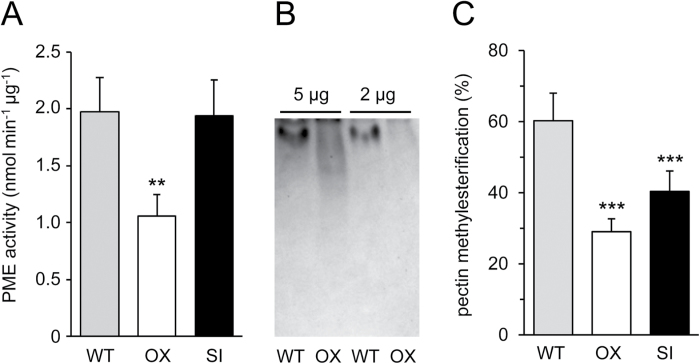
Effect of SBT3 expression on PME activity and pectin composition. (A) PME activity was analysed in leaf extracts of WT (grey), *SBT3-OX* (white) and *SBT3-SI* plants (black) and is expressed as nanomoles methanol released per minute and microgram protein. Each data point represents the mean of at least three biological replicates, with six independent measurements per data point. Two and three independent transgenic lines were analysed for *SBT3-OX* (G18, G19) and *SBT3-SI* (SI-12, SI-14, SI-21), respectively. PME activity differs significantly in *SBT3-OX* as compared with *SBT3-SI* and WT plants at *P*=0.004 (one-way ANOVA with Tukey’s *post hoc* test for multiple comparisons). (B) Protein extracts from WT and *SBT3-OX* (line G18) plants (2 and 5 µg of total protein) were separated by isoelectric focusing. Ruthenium red staining of de-methylesterified pectin was used to detect PME activity with citrus pectin as the substrate. (C) The degree of pectin methylesterification (DM) was analysed in 6-week-old tomato plants and compared with WT (grey), *SBT3-OX* (white) and *SBT3-SI* plants (black). At least three biological replicates were analysed for WT, for each of the three independent *SBT3-SI* lines (SI-12, SI-14, SI-21), and four *SBT3-OX* lines from two independent transformation events (G18, G19). Data represent the mean±standard deviation. Asterisks indicate statistically significant differences from WT at *P*<0.001 (*t*-test).

These findings prompted us to investigate whether the effect of SBT3 expression levels on *M. sexta* performance (Fig. 1B) can be explained by SBT3-mediated changes in PME activity and/or cell wall composition. An analysis of neutral and acidic sugar composition did not reveal any differences between wild-type and *SBT3* transgenics (see Supplementary Fig. S4). PME activity, however, was substantially reduced in the foliage of *SBT3-OX* plants as compared with wild-type and *SBT3-SI* plants (Fig. 5A). Apparently, SBT3 is not required for PME maturation or secretion, but may rather be involved in the degradation of PMEs and down-regulation of PME activity. Indeed, in-gel assays of PME activity indicated that a major PME isoform is missing in *SBT3-OX* plants (Fig 5B).

Unexpectedly, the loss of this particular PME isoform in *SBT3-OX* plants did not result in a corresponding increase in the degree of HG methylesterification (DM), but rather in a lower DM in *SBT3-OX* as compared with wild-type plants (Fig 5C). This apparent discrepancy may be due to the compensatory induction of other PME isoforms, the downregulation of PME inhibitors, or compensatory changes in pectin methyltransferase activity. Such a compensatory response to interference with PME activity has previously been described in Arabidopsis roots (Wolf *et al.*, 2012). Since our activity assay only detected PMEs that are active at pH 7.5 and on highly methylesterified substrate, a possible compensatory increase of other PME activities may have escaped detection. Similar to *SBT3-OX* plants, a reduction in DM was also observed in plants silenced for *SBT3* expression (Fig. 5C). The effects of SBT3 expression on pectin methylesterification thus appear to be complex and cannot be explained by SBT3-mediated processing of a single PME. To explain the observed effects, a better understanding of the multiple PME isoforms, their interaction with PME inhibitors and processing by SBTs, and the contribution of other homogalacturonan-modifying enzymes would be required, which, however, is beyond the scope of the present study. Nonetheless, because the performance of *M. sexta* larvae is obviously not correlated to the observed changes of PME activity or DM in *SBT3-OX* and *-SI* plants, it can be concluded that SBT3-mediated changes in cell wall structure or composition are not causally linked to insect resistance.

Alternatively, the apparent effects of SBT3 on PME activity (Fig. 5A) and pectin structure (Fig. 5C) may be relevant during certain stages of plant development. SBT3 is strongly expressed in the micropylar endosperm (Fig. 3A, B) where PME activity controls the resistance of the endosperm to the protruding radicle (Muller *et al.*, 2013; Scheler *et al.*, 2015), and where a reduction of PME activity by SBT3 may facilitate endosperm rupture and completion of germination. Irrespective of a potential role for SBT3-mediated changes in PME activity during germination, the effect of SBT3 expression on insect resistance of *SBT3-OX* and *-SI* plants remains unexplained and cannot be attributed to PME-mediated changes in pectin composition. We therefore addressed the possibility that SBT3 may exert its effect on larval performance only after ingestion, within the digestive system of the insect.

### Post-ingestive activity of SBT3

The nutritional quality of foliage is a limiting factor for herbivore growth and development. A major defense strategy of plants thus aims to restrict the insect’s ability to digest dietary protein and to retrieve essential nutrients (Felton, 2005). In addition to proteinase inhibitors targeting the digestive proteinases of the insect (Jongsma and Beekwilder, 2008), jasmonate-inducible anti-nutritive proteins include arginase and threonine deaminase, which are highly active in the midgut of *M. sexta* where they degrade the essential amino acids arginine and threonine, respectively (Chen *et al.*, 2005, 2008). Also part of the jasmonate-inducible defense arsenal are polyphenol oxidase, catalysing the oxidation of phenolics to form electrophilic quinones that may bind to the nucleophilic amino acid side chains of dietary protein, and acid phosphatase (Vegetative Storage Protein 2) with an as yet unknown function in anti-nutritive defense (Liu *et al.*, 2005; Constabel and Barbehenn, 2008; Bosch *et al.*, 2014*a*). All these proteins were found to be stable in the harsh environment of the digestive system, which in *M. sexta* and other caterpillars is characterized by highly alkaline pH (pH 8–11) and an abundance of proteolytic activities (Felton, 2005). The defensive function of these proteins relies on their stability and the ability to retain activity under such adverse conditions (Chen *et al.*, 2005; Chi *et al.*, 2011).

**Fig. 6.**
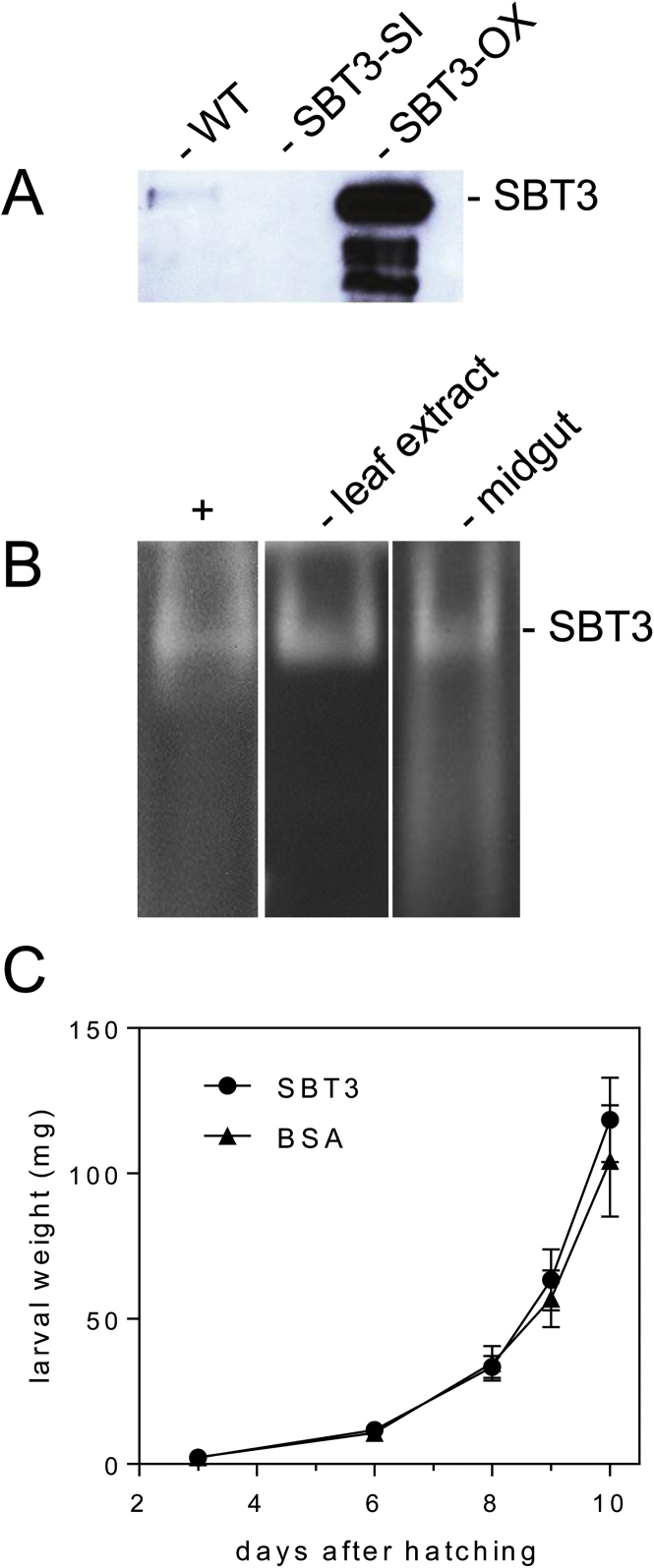
Post-ingestive activity of SBT3. (A) Stability of SBT3 in the digestive system of *M. sexta* larvae. Larvae of *M. sexta* were raised on WT, *SBT3-OX* (G2) or *SBT3-SI* (SI-21) plants. Protein extracts from frass (10 µg total protein) of fifth-instar larvae were separated by SDS-PAGE and analysed on western blots using a polyclonal antiserum against SBT3. (B) Activity of SBT3 in the digestive system of *M. sexta* larvae. Protein extracts from *SBT3-OX* plants (20 µg) and *M. sexta* midgut (20 µg) and purified SBT3 protein (15ng; positive control, +) were separated by acidic PAGE on 6.75% gels with 0.5% co-polymerized gelatine. Gelatinolytic activity of SBT3 is visualized by Coomassie staining as a clear band against a dark background. (C) Growth of *M. sexta* larvae raised on artificial diet supplemented with 100 µg g^–1^ fresh weight of SBT3 (circles) or BSA (triangles). Larval weight was determined after 3 (*n*=37), 6 (*n*=32), 8 (*n*=23), 9 (*n*=20), and 10 (*n*=18) days. Data represent the average weight of all larvae alive at the respective time point±standard error.

The exceptional stability of SBT3 and its activity at alkaline pH (60% at pH 11; Cedzich *et al.*, 2009; Ottmann *et al.*, 2009) prompted us to investigate whether SBT3 is stable and active in the midgut of *M. sexta* larvae. Undigested SBT3 protein was detected in the feces of larvae that were raised on *SBT3-OX* plants (Fig. 6A). A smaller amount of apparently intact SBT3 was also observed when insects fed on wild-type plants, whereas SBT3 was undetectable when insects were raised on *SBT3-SI* transgenics. In-gel proteinase assays further confirmed that SBT3 is active in the larval midgut (Fig. 6B). The data suggest that SBT3 may serve a post-ingestive function in plant defense by degradation or processing of proteins in the insect digestive system.

A post-ingestive role in plant defense was previously shown for cysteine proteinases in maize (Pechan *et al.*, 2000 and papaya (Konno *et al.*, 2004), LAP in pigeon pea (Lomate *et al.*, 2013), and has also been suggested for LapA in tomato (Gu *et al.*, 1999; Chen *et al.*, 2007). Their mechanism of toxicity is unknown, except for the maize papain-like cysteine proteinase Mir1-CP. Mir1-CP disrupts the peritrophic membrane, a chitin matrix that lines the midgut epithelium, assists in digestive processes, and protects the caterpillar midgut from physical and chemical damage (Pechan *et al.*, 2002; Lopez *et al.*, 2007; Fescemyer *et al.*, 2013). Physiological substrates, however, remain to be identified for Mir1-CP and the other proteases alike.

Potential SBT3 substrates may include insect proteins as well as proteins from tomato foliage that are ingested together with SBT3. Several plant defense proteins were in fact shown to require proteolytic processing in order to perform their defensive role, including threonine deaminase, polyphenol oxidase, and urease (Ferreira-DaSilva *et al.*, 2000; Wang and Constabel, 2004; Chen *et al.*, 2007; Gonzales-Vigil *et al.*, 2011; Stanisçuaski and Carlini, 2012). Similarly, elicitor peptides have been identified that are proteolytically derived from dietary protein when the plant is attacked by insects (Schmelz *et al.*, 2007; Pearce *et al.*, 2010). These peptides serve as damage-associated molecular patterns allowing for non-self recognition and subsequent activation of plant defense. The proteases required for the formation of these peptides have not been identified and could be of either plant or insect origin.

To address the question whether SBT3 is directly toxic to the insect, targeting proteins of the larval digestive system, or whether it may contribute to the processing of defense-related plant proteins, we performed feeding assays with artificial diet supplemented with SBT3 in amounts exceeding the concentration in wild-type leaves by two- to three-fold. BSA was added as a control. Larvae of *M. sexta* were indifferent to the presence of SBT3 in their diet, showing equal growth on SBT3- and BSA-supplemented media (Fig. 6C). Any direct toxic effect of SBT3 is thus unlikely. This conclusion is consistent with the observation that *SBT3-OX* plants with dramatically increased SBT3 levels (Fig. 6A) have little impact on larval growth. This leaves the possibility that SBT3 acts on plant proteins that are ingested together with SBT3 either to modulate their anti-nutritive activity, or to release peptides that may act as elicitors of plant defense. These substrates of SBT3 and any signals derived from them remain to be identified.

## Conclusions

The expression of *SBT3* was found to be induced in damaged leaves but not systemically in response to wounding and insect attack. The time course of *SBT3* induction resembled that of late wound response genes suggesting a role for SBT3 in the downstream defense response. Using transgenic plants with altered SBT3 expression levels we confirm that SBT3 contributes to insect resistance in tomato plants. Improved performance of *M. sexta* larvae on SBT3-deficient plants may be explained in part by the systemic defense response that was found to be attenuated when *SBT3* was silenced. SBT3 is thus implicated in the late wound signaling pathway contributing to the induction of defense genes in unwounded tissues.

As an alternative or additional mode of action, SBT3 may play a post-ingestive role in plant defense. Facilitated by its exceptional stability and high activity at alkaline pH, SBT3 may exert its defensive function in the insect’s digestive system. SBT3 target proteins, possibly including tomato proteins that are ingested along with the protease, remain to be identified. While SBT3 was also found to affect PME activity and the level of pectin methylesterification, these effects were unrelated to its role in herbivore defense.

## Supplementary data

Supplementary data are available at JXB online.

Supplementary methods.

Figure S1. Generation of *SBT3-OX* and *SBT3-SI* plants

Figure S2. qRT-PCR and western blot analysis of SBT3 expression in *SBT3-OX* and *SBT3-SI* plants.

Figure S3. Southern blot analysis of SBT3pro:GUS reporter lines.

Figure S4. Analysis of cell wall neutral and acidic sugar composition.

## Funding

This research was supported by a grant [SCHA 591/4-1] of the German Research Foundation (DFG) to AS.

## Supplementary Material

Supplementary Data

## Abbreviations:

DM: degree of methyl esterification
GUS: β-glucuronidase
HG: homogalacturonan
JA: jasmonate
OPR: oxophytodienoate reductase
ORF: open reading frame
LAP: leucine aminopeptidase
LOX: lipoxygenase
OX: overexpression
PI-II: proteinase inhibitor II
PME: pectin methylesterase
PPO: polyphenol oxidase
S1P: site-1-protease
SBT: subtilase
SI: silenced.
