# Supplementary material for: The subtilisin-like protease SBT3 contributes to insect resistance in tomato
Source: J Exp Bot. 2016 Jun 3;67(14):4325–38. doi: 10.1093/jxb/erw220 (PMC5301937; doi:10.1093/jxb/erw220)
Supplement: Supplementary Data [file supp_67_14_4325__index.html]

The subtilisin-like protease SBT3 contributes to insect resistance in tomato — The subtilisin-like protease SBT3 contributes to insect resistance in tomato — Supplementary Data 

# The subtilisin-like protease SBT3 contributes to insect resistance in tomato

## Supplementary Data

Data files

- Supplementary\_table\_S1\_figures\_S1\_S4.pdf - Supplementary Data
