# Supplementary material for: The subtilisin-like protease SBT3 contributes to insect resistance in tomato
Source: J Exp Bot. 2016 Jun 3;67(14):4325–38. doi: 10.1093/jxb/erw220 (PMC5301937; doi:10.1093/jxb/erw220)
Supplement: Supplementary Data [file supp_erw220_Supplementary_table_S1_figures_S1_S4.pdf]

The subtilisin-like protease SBT3 contributes to insect resistance in tomato

Michael Meyer, Franziska Huttenlocher, Anja Cedzich, Susanne Procopio, Jasper Stroeder, Corinne Pau-Roblot, Michelle Lequart-Pillon, Jérôme Pelloux, Annick Stintzi and Andreas Schaller

Primer Sequences

1. PCR-primers. All primers were obtained from Operon, Cologne, Germany

cloning of the SBT3 hairpin construct for RNAi (SBT3-SI)

(XbaI/XhoI and HindIII/KpnI restriction sites underlined)

SBT3-SI, forward: 5'- GGGTCTAGACTCGAGGTTATTTGAATCAGTAGTGT -3'
SBT3-SI, reverse: 5'- GGGAAGCTTGGTACCAATCAATAGTGGAAGAATGC-3'

cloning of the SBT3 overexpression construct (SBT3-OX)

(SmaI/PstI restriction sites underlined, start and stop codons in bold italics)

SBT3-OX, forward (3DH5): 5'- GGCCCGGG***ATG***GAGTTACTTCATCTT -3'
SBT3-OX, reverse (3DH3): 5'- GGCTGCAG***TTATTA***CCAGACCTCAAT-3'

Cloning of the SBT3 promoter:GUS construct:

(SalI and BamHI restriction sites underlined, start codon in bold italics)

SBT3p, forward: 5'-CCGTCGACCTTCTTCTTCTTCAAATTGTTCC–3'

SBT3p, reverse: 5'-CCGGATCC***CAT***TGTAGATCTTTCTGTTGAGT–3'

Primers for the generation of radioactive probes

SBT3-probe, forward: 5`-ACTCCTCAAGATTACGTAAATCTCC-3`

SBT3-probe, reverse: 5`-CAATAATAGGAGATGTTACTATCGGAC-3`

5PI-II, forward: 5`-GGGAATTCGTCGCTTACCTACTAATAGTTC-3`

3PI-II, reverse: 5`-GGGAATTCACACAACTTGATGCCCACATTA-3`

NPTII, forward 5'- AAGAAGGCGATAGAAGGCGAT –3'

NPTII, reverse 5'- TATGACTGGGCACAACAGAC –3'

Primers for qRT-PCR analysis

target gene	accession number	primer sequence (5'→ 3')	concentration (nM)	product size (bp)
Elongation Factor 1α (Ef1α)	X14449	FOR: AGCCCATGGTTGTTGAGACCTTTG<br>REV: TTCGAAACACCAGCATCACACTGC	100	190
Ubiquitin (UBI3)	X58253	FOR: CTCTTGCCGACTACAACATCCA<br>REV: AGCACCGCACTCAGCATTA	200	221
Tomato Subtilase 3 (SBT3)	AJ006376	FOR: CTCGAGCAAGACTTAAAGCAGC<br>REV: GGAACGTGATGGTTGCTGTG	100	162
12-Oxophytodien-oate reductase 3 (OPR3)	AJ278332	FOR: TCTCCGACTTCAGCTGGGTTTC<br>REV: ATGCACGACCAACATGCCACAG	100	139
Lipoxygenase (LoxD)	U37840	FOR: GGCCGTGGTTGACACATTATC<br>REV: GCCTGAACTTGGTGCCAATAG	100	230
Proteinase Inhibitor 2 (PI-II)	K03291	FOR: GGATATGCCCACGTTCAGAAGGAA<br>REV: AATAGCAACCCTTGTACCCTGTGC	100	257
Leucine aminopeptidase A (LapA)	U50151	FOR: CCTGGTAATGGCGGTGCTAT<br>REV: GCACCCATTCCACCAGAGTT	100	157

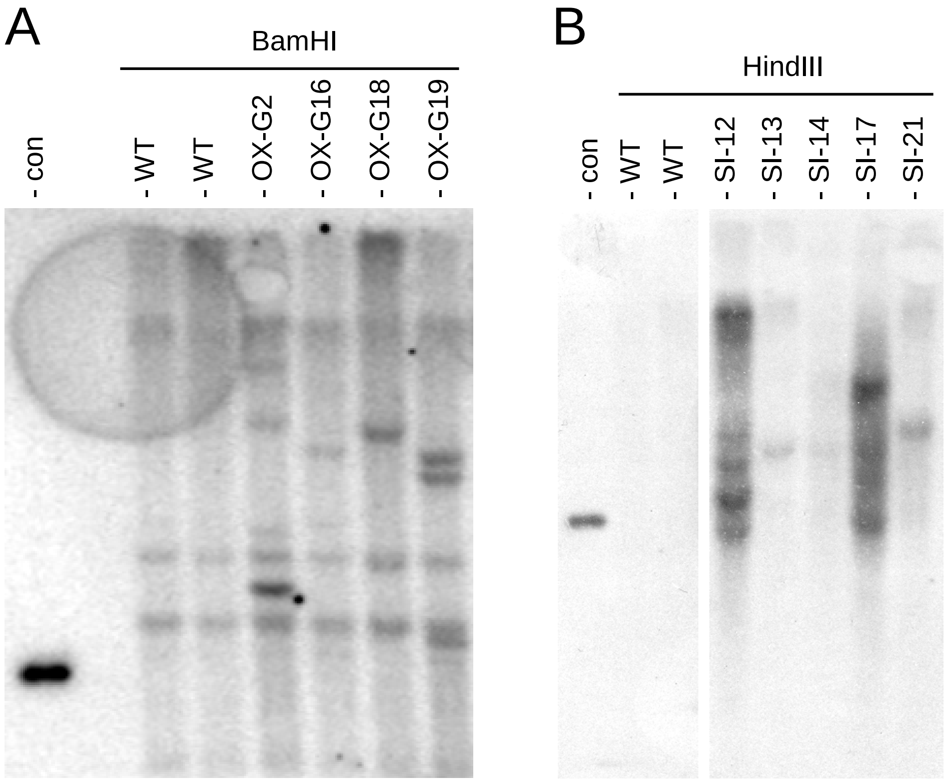

Figure S1. Southern Blot analysis of *SBT3-OX* (**A**) and *SBT3-SI* plants (**B**). Ten µg genomic DNA from wild-type (WT) and transgenic *SBT3-OX* and *SBT3-SI* lines were digested with *Bam*HI (**A**) or *Hind*III (**B**) separated by electrophoresis on 0.8 % agarose gels and blotted to nitrocellulose membranes. 100 pg of the linearized transformation vector pART27 were used as positive control (con). The membranes were hybridized to a radio-labelled probe (RadPrime labelling kit; Invitrogen, Karlsruhe, Germany) corresponding to the *nptII* selectable marker gene. The membranes were washed at high stringency (30 min at 0.2 x SSC, 0.5 % SDS, 60 °C), and analyzed on a phosphoimager (Typhoon Trio; GE Healthcare, Freiburg, Germany). Band patterns differ for all lines except SI-13 and SI-14, indicating independence of transformation events.

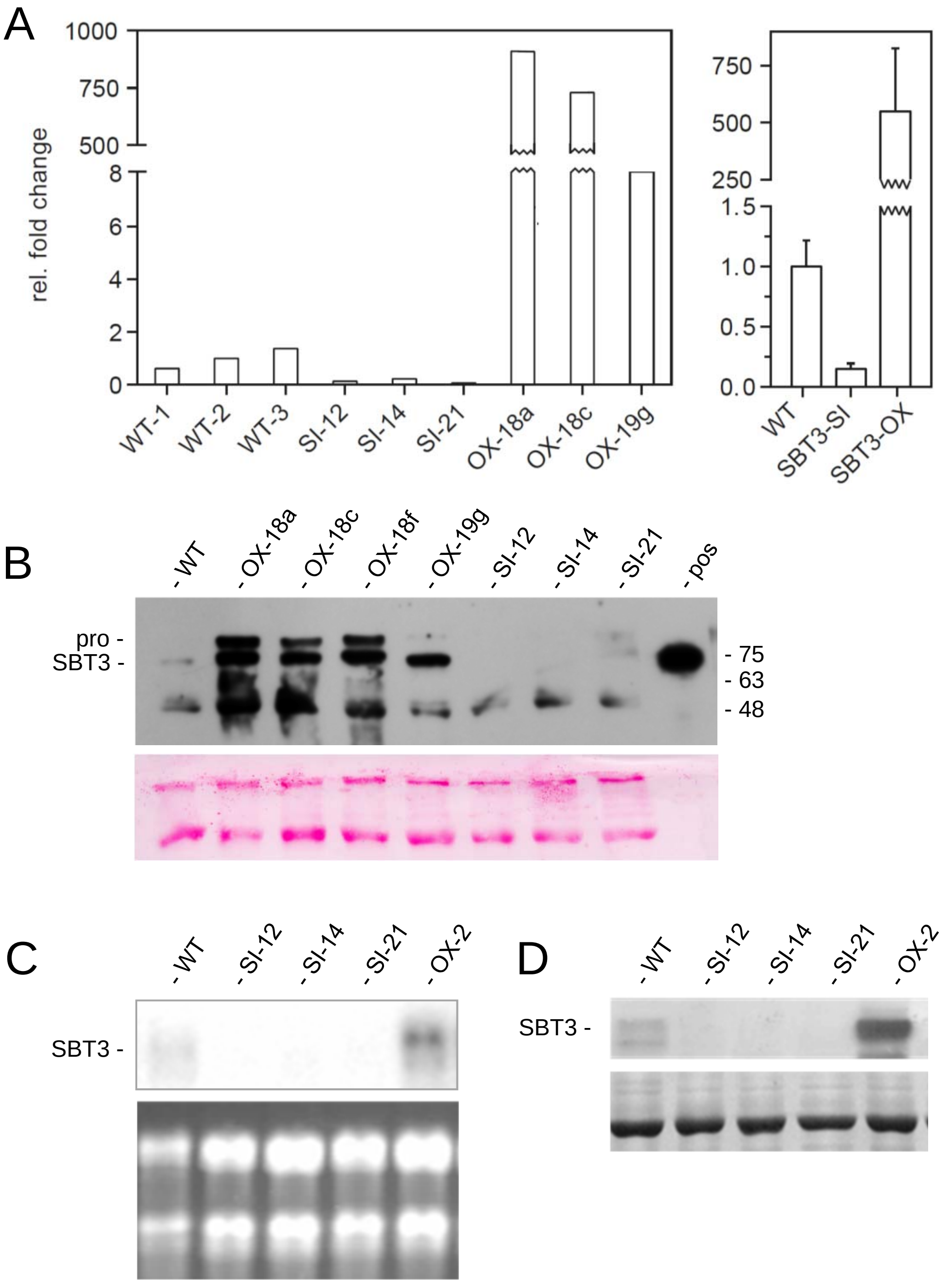

Figure S2. qRT-PCR (**A**), western (**B,D**) and northern blot analysis (**C**) confirming over-expression and silencing of SBT3 in *SBT3-OX* and *SBT3-SI* plants, respectively. (**A**) Total RNA was isolated from five pooled leaves of wild type (WT), *SBT3-SI* (lines SI-12, SI-14, and SI-21) and *SBT3-OX* plants (lines OX-18a, OX-18c and OX-19g) and analysed for SBT3 expression by qRT-PCR. Transcript abundance was normalized against *UBI3* and *EF-1α* expression, and is given relative to the *SBT3* level in a pool of 20 unwounded wild-type plants. The left panel shows the results for each of the three tested lines individually, the right panel shows the mean of the three lines +/- standard error. (**B, D**) 20 µg total leaf protein from WT, and *SBT3-OX* and *SBT3-SI* transgenic lines were separated by SDS-PAGE and transferred to a nitrocellulose membrane. 20 ng purified SBT3 protein was used as positive control. Blots were developed using a polyclonal anti-SBT3 serum and horseradish peroxidase-coupled goat-anti-rabbit IgG as the secondary antibody followed by enhanced chemiluminescence (ECL) detection of peroxidase activity. The blot stained with Ponceau S and a duplicate Coomassie-stained gel are shown as loading controls in **B** and **D**, respectively. (**C**), northern blot analysis was performed with total RNA isolated as in **A**. The blot was hybridized with a radiolabelled fragment of the SBT3 cDNA. A duplicate ethidium bromide-stained gel is shown as a control for RNA integrity and loading.

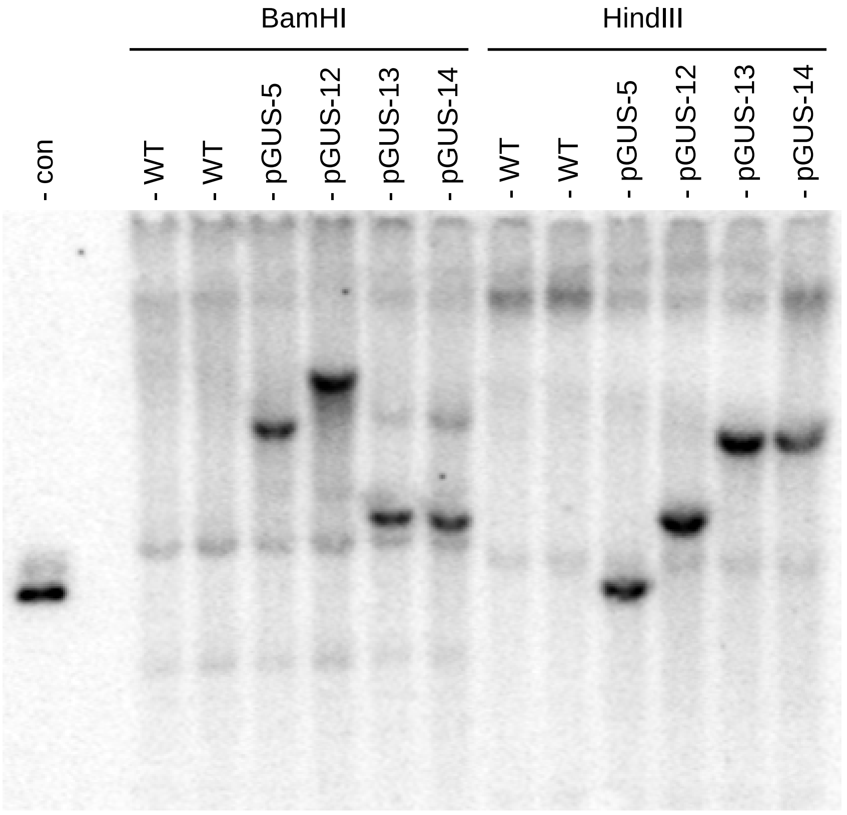

Figure S3. Southern Blot analysis of SBT3pro:GUS reporter lines. Ten µg genomic DNA from wild-type (WT) and transgenic tomato plants carrying a *SBT3* promoter-reporter (GUS/*uidA*) construct were digested with *Bam*HI or *Hind*III, separated by electrophoresis on 0.8 % agarose gels and blotted to a nitrocellulose membrane. 100 pg of the linearized transformation vector pART27 were used as positive control (con). The membrane was hybridized to a radiolabelled probe (RadPrime labelling kit; Invitrogen) corresponding to the *nptII* gene. The membranes were washed at high stringency (30 min at 0.2 x SSC, 0.5 % SDS, 60 °C), and analyzed on a phosphoimager (Typhoon Trio; GE Healthcare). Different bands in the individual transgenic lines indicate independent transformation events for lines 5, 12, and 13.

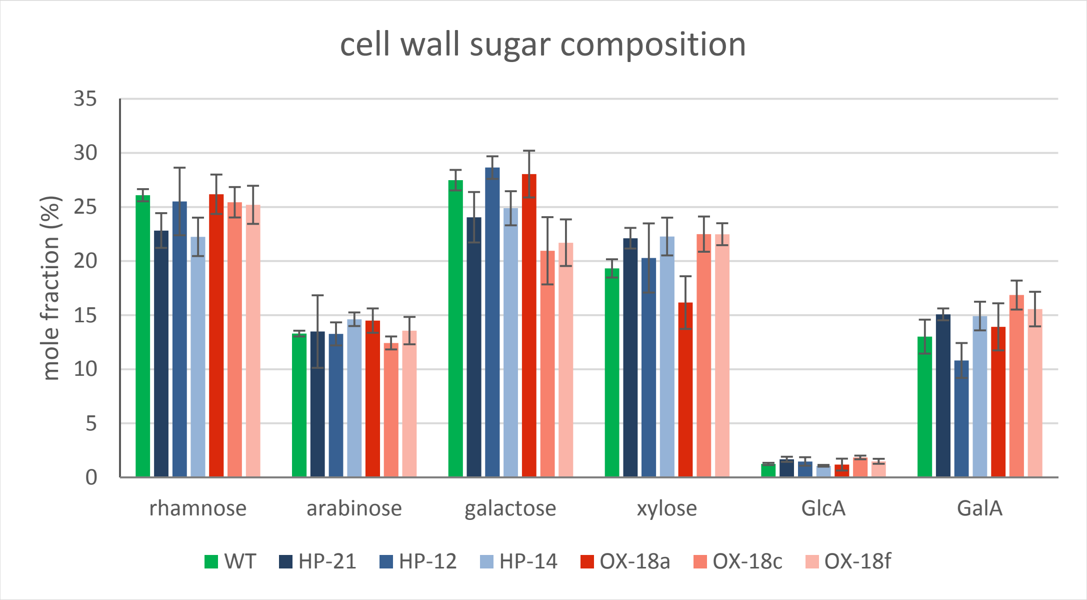

Figure S4. Neutral and acidic sugar composition of cell walls of *SBT3-OX*, *SBT3-SI* and wild-type tomato plants. Neutral and uronic sugars were analyzed in cell wall hydrolysates of wild-type (WT), *SBT3-SI* and *SBT3-OX* plants by High Performance Anion Exchange Chromatography (HPAEC). Data represent the mean +/- standard deviation of six biological replicates, each analysed in duplicate for WT, *SBT3-OX* (lines 18a, 18c and 18f), and *SBT3-SI* (lines 12, 14, and 21).
